# A Case of Aplasia Cutis Congenita in the Setting of Maternal Carbimazole Use in the First Trimester

**DOI:** 10.1210/jcemcr/luad130

**Published:** 2023-11-16

**Authors:** Colin McGrath, Nancy O’Hanrahan, Michael Conall Dennedy, Michael A Boyle

**Affiliations:** Department of Neonatology, Rotunda Hospital, Dublin, D01 P5W9, Ireland; Department of Neonatology, Rotunda Hospital, Dublin, D01 P5W9, Ireland; Department of Endocrinology, University Hospital Galway, Galway, H91 YR71, Ireland; Department of Neonatology, Rotunda Hospital, Dublin, D01 P5W9, Ireland; Department of Neonatology, Children's University Hospital, Dublin, D01 XD99, Ireland

**Keywords:** cutis aplasia, hyperthyroidism, carbimazole, Graves’ disease

## Abstract

Aplasia cutis congenita (ACC) is one of several congenital malformations associated with antithyroid/thiourylene drug use in pregnancy. While uncommon among the general population (1-3/100 000 cases), the risk among those on thiourylenes is between 1.6% and 3%. The scalp is the most common site for this congenital anomaly.

We present the case of a male infant with multifocal ACC of the scalp discovered at birth and born to a mother with Graves disease that was controlled during pregnancy using carbimazole. Thyroid function tests were normal throughout the pregnancy. There was no involvement of underlying subcutaneous tissue or structures. At age 18 months, the single largest lesion remained with only partial coverage. Prospective management involved periodic surveillance with planned 2-stage repair.

This case reinforces the association between the antithyroid drugs carbimazole (CMZ) and methimazole (MMI) and supports the proposition of an MMI/CMZ embryopathy. It adds to a literature of case reports in which malformations arise in offspring of such mothers whose thyrotoxicosis is controlled antenatally, thereby challenging the suggestion that ACC is attributable to poorly controlled disease rather than thiourylenes. As yet the underlying mechanism is not understood, nor is it known why MMI and CMZ may cause potentially significant embryopathy while congenital defects attributable to the structurally similar propylthiouracil are typically less severe.

## Introduction

Aplasia cutis congenita (ACC) or aplasia cutis is a rare skin defect characterized by either a focal or extensive absence of the epidermis, dermis, and in some cases the subcutaneous tissue. It may be unifocal or multifocal and involve underlying bone, intracranial, or vascular structures. Although ACC may affect any part of the body, the scalp is most commonly affected.

Aplasia cutis was among the first congenital defects attributed to methimazole in 1972 [1]. In the following years, methimazole (MMI) and its prodrug carbimazole (CMZ) were increasingly associated with several congenital malformations including choanal atresia, tracheoesophageal fistula, and patent vitello-intestinal duct [2]. Recent clinical reviews have supported an increased incidence of congenital defects in those exposed to these drugs in utero [3]. In one retrospective study of the Danish Birth and Danish Medical registries, the use of antithyroid medication in pregnancy was associated with birth defects in 3% to 4% of exposed infants [4]. Here we report a rare case of aplasia cutis congenita associated with maternal use of CMZ for Graves hyperthyroidism in pregnancy.

## Case Presentation

A term male infant was born via spontaneous vaginal delivery to a gravida 4, para 3 White mother of a nonconsanguineous couple. The infant's mother was diagnosed with Graves disease following the birth of her third child a number of years before and was maintained on CMZ 5 mg daily as her endocrine team were unable to successfully wean her from it and scheduled definitive treatment was postponed due to unplanned pregnancies. His mother was not aware of the importance of contacting her endocrine team for preconceptual planning or on becoming pregnant and as such was not identified as an issue until a review at a maternity clinic appointment at 20 weeks.

As she was beyond the first trimester and previous efforts to wean from CMZ had failed, a pragmatic approach was adopted by which she continued unchanged with careful monitoring thereafter. While adherence prior to pregnancy was previously poor, her dose was unchanged and she remained adherent to treatment during this index pregnancy. Antenatal testing confirmed that the patient remained euthyroid on antithyroid medication throughout gestation (Table 1).

**Table 1.** Maternal thyroid function tests during the course of pregnancy

	34-wk gestation	28-wk gestation	20-wk gestation
TSH	0.21 (0.1-4.0 mIU/L)	0.42 (0.1-4.0 mIU/L)	0.51 (0.1-4.0 mIU/L)
Free T4	16.8 (12-22 pmol/L) 1.31 (0.93-1.71 ng/dL)	15.4 (12-22 pmol/L) 1.2 (0.93-1.71 ng/dL)	16.8 (12-22 pmol/L) 1.31 (0.93-1.71 ng/dL)
Free T3			4.9 (3.1-6.8 pmol/L) 3.18 (2.0-4.42 pg/mL)
TRAb			<0.8 (0-1.8 IU/L)

Système International (SI) unit ÷ conversion factor (CF) = conventional unit (free T4 CF 12.87, free T3 CF 1.54).

Abbreviations: T3, 3,5,3′-triiodothyronine; T4, thyroxine; TRAb, thyrotropin receptor antibody; TSH, thyrotropin.

The child was vigorous at delivery with APGAR scores of 9 at 1 minute and 10 at 5 minutes and weighing 3.75 kg with head circumference of 35.9 cm. Six scalp lesions were observed at the vertex comprising absent skin and subcutaneous soft tissue. These were of varying size, the largest of which measured 26 mm × 5 mm and 6 mm × 4 mm and were well demarcated. The examination was otherwise normal. Clinical photography was obtained and a dermatology opinion confirmed the diagnosis of ACC.

## Diagnostic Assessment

**Figure 1.**
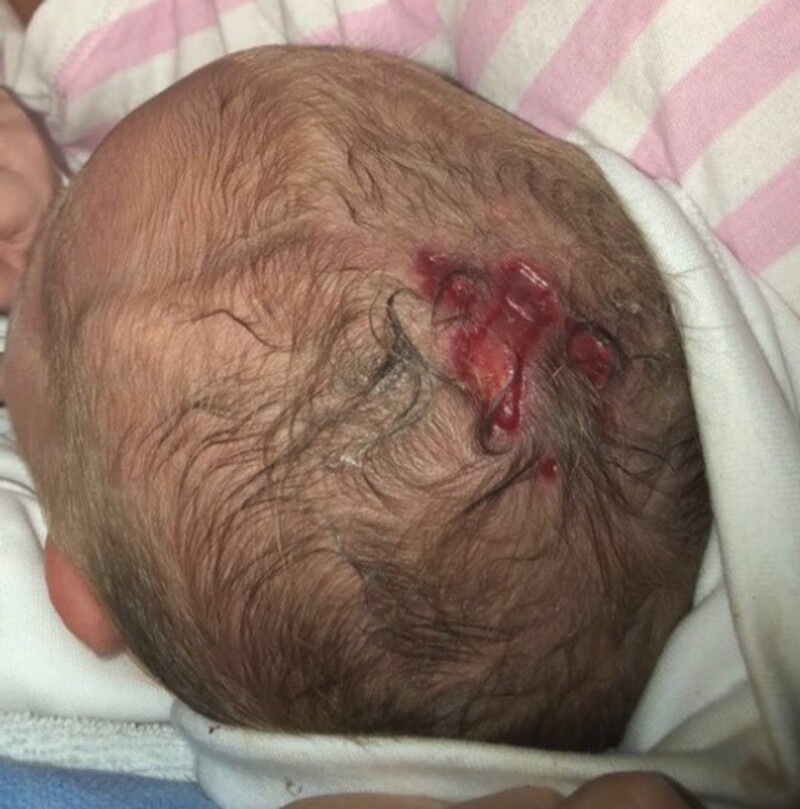
Multiple aplastic skin lesions at the vertex present at birth.

Clinical photography was taken (Fig. 1). Swabs of the lesions were sent for culture and antibiotics were commenced empirically. Given the maternal history of hyperthyroidism, thyroid function testing was performed on day 10. This revealed a thyrotropin level of 4.1 mIU/L (reference range, 0.1-5.5 mIU/L) and free thyroxine (T4) of 18.3 pmol/L (reference range, 10-36 pmol/L)/1.42 (reference range, 0.78-2.78 ng/dL), which was normal. A cranial ultrasound on day 2 revealed no associated cranial neural tube defect. A cranial vault computed tomography scan at age 4 months was essentially normal and confirmed that there was no underlying bone involvement or suture synostosis.

## Treatment

No specific treatment was required during the infant's inpatient stay. A referral to plastic surgery was made for further management.

## Outcome and Follow-up

**Figure 2.**
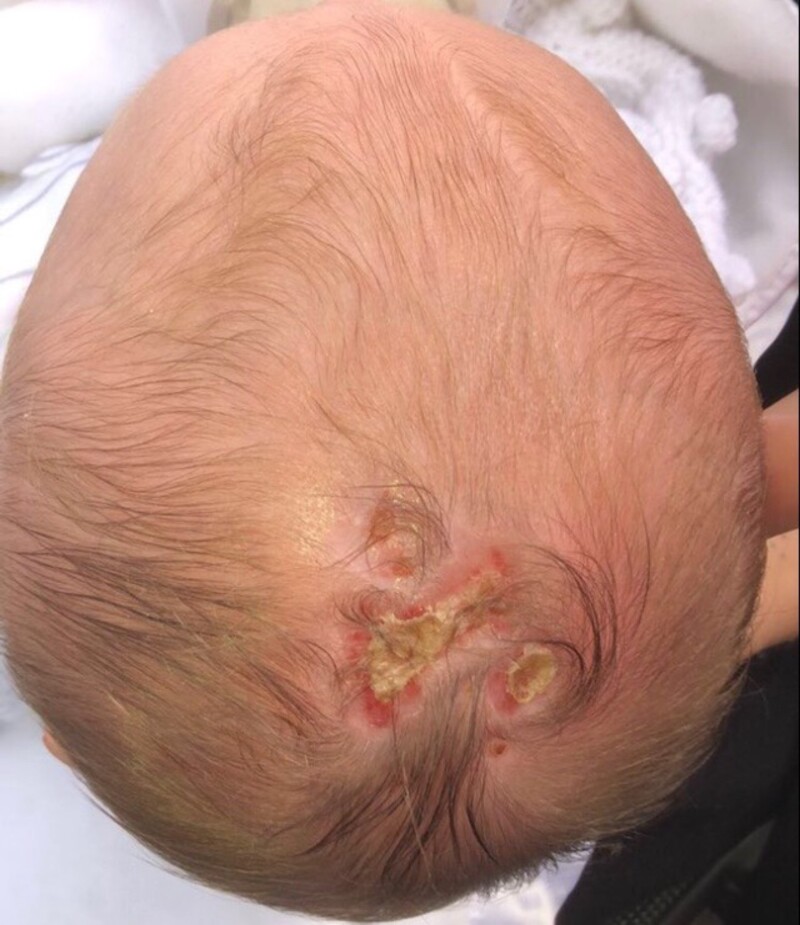
Partial granulation of aplasia cutis congenita at 6-week checkup.

Partial resolution of the scalp lesions was evident at 6-week review (Fig. 2). There was almost complete coverage of the 4 smaller lesions and partial coverage was attained for the 2 largest defects. The plastic surgery team elected against surgery in the immediate setting to allow a period of time to allow healing by secondary intention. On review at age 18 months, a single lesion persisted at the vertex measuring 3 × 4 cm and a plan was made for 2-stage repair, the first stage at age 5 years. The aim of the surgical intervention was to fully excise the bald patch with the final resection planned for during adolescence. Interestingly, on the subsequent fifth pregnancy for this woman CMZ was stopped at 5 weeks’ gestation on receipt of a positive pregnancy test and changed to propylthiouracil (PTU). She was asymptomatic and euthyroid prior to transfer to PTU. On review in the second trimester at 14 weeks’ gestation, her free T4 was rising (14.3, 17.2, 21.3 mIU/L)/(1.11, 1.34, 1.66 ng/dL) and she was changed back to CMZ. She was asymptomatic throughout the pregnancy. A female infant was born at full term, well and with no ACC on examination. The woman later had definitive surgical treatment for Graves disease.

## Discussion

ACC is an uncommon condition affecting 1 to 3 per 100 000 live births. Ninety percent of defects arise on the scalp, 15% to 30% of which will be further complicated by underlying skull or soft tissue aplasia/hypoplasia. While the majority comprise isolated defects, approximately 30% will involve multiple lesions. Although ACC is usually an isolated phenomenon without a clear cause, it may be observed in some chromosomal anomalies such as heterozygous mutation in the *BMS1* gene on chromosome 10q11 [5].

While it has been reported that antithyroid drug–induced agranulocytosis is associated with HLA-B*27:05 and with other single-nucleotide variations, there are no published data linking specific genetic loci with ACC risk in the setting of antithyroid medication use. Familial cases have also been reported with both autosomal dominant and recessive inheritance patterns, with dominant being more commonly seen.

It has also been reported alongside other congenital abnormalities including cardiac lesions, gastrointestinal (gastroschisis and exomphalos), and central nervous system anomalies including spina bifida and spinal dysraphism. A strong association between ACC and maternal exposure to CMZ/MMI has been the subject of several case reports and studies over the past 40 years. This entity has been termed *MMI/CMZ embryopathy* and includes presentations of isolated phenomena or more severe embryopathy including multiple abnormalities such as ACC, choanal atresia, omphalocele, and esophageal atresia.

There has historically long remained a lack of support for such association from medium-sized studies. Between 1987 and 1994, 3 such studies evaluating outcomes of infants exposed to MMI in utero, ranging from 24 to 243 participants, did not observe any clear association with neonatal skin defects [6, 7]. A follow-up prospective analysis of 241 pregnancies across 10 European teratology registers identified no increased risk of congenital anomaly in exposed infants [8].

Given the low incidence of ACC in the general population and an increasingly frequent relationship highlighted in epidemiological studies and case reports, it was thought that much larger studies would be required to support causality between antithyroid drugs and such neonatal skin defects. Consequently, 2 large-scale retrospective studies and a meta-analysis of a further 6 cohort studies demonstrated that exposure to MMI or CMZ in utero increased the risk of ACC and other congenital anomalies by 64% [1, 4].

There is also animal evidence to support a causal association of exposure to thionamides and aplasia cutis. The absolute risk of MMI/CMZ embryopathy following first-trimester antithyroid drug exposure is now thought to be 1.6% to 3%.

Several authors have postulated that it is underlying maternal thyrotoxicosis during pregnancy that is the basis for such defects. Our case of ACC in an otherwise euthyroid mother adds to more than 31 previous case reports in which there was no evident thyrotoxicosis in presumed MMI/CMZ embryopathy [9].

Professional bodies and other authors have long advised on the importance of preconceptual planning and alternative antithyroid treatment in pregnancy [10]. Experts advocate a PTU-first approach particularly during the essential period of organogenesis during the first trimester, which may later be followed by a return to MMI/CMZ in midpregnancy when initial organ development is complete.

Although a definitive interaction between MMI/CMZ and congenital anomalies like ACC has not yet been established, the association is strong. With the concept of MMI/CMZ embryopathy now established, a direct causal mechanism must be sought and larger epidemiological studies are required to further quantify the risk of congenital malformation among mothers on antithyroid medication.

## Learning Points

- There is a strong association between the antithyroid drugs MMI and CMZ with ACC.
- A clinical constellation of anomalies exists, attributable to these medications (termed *MMI/CMZ embryopathy*).
- This relationship is apparently independent of maternal thyroid status.
- PTU should be used periconceptually and in the first trimester to minimize risk of anomaly during organogenesis.
- It is important to counsel women of reproductive age on the importance of liaising with their endocrinology team when planning a family or becoming pregnant while on these medications

## Contributors

N.O'H. and M.A.B. (the patient's primary physician) proposed the topic of this case report. C.M. and N.O'H researched and wrote the paper. The article was reviewed and edited by M.A.B. and M.C.D.

## Data Availability

Data sharing is not applicable to this article as no data sets were generated or analyzed during the current study.

## Abbreviations

ACC: aplasia cutis congenita
CMZ: carbimazole
MMI: methimazole
PTU: propylthiouracil

## References

[1] Andersen SL, Olsen J, Wu CS, Laurberg P. Birth defects after early pregnancy use of antithyroid drugs: a Danish nationwide study. J Clin Endocrinol Metab. 2013;98(11):4373‐4381. 24151287 10.1210/jc.2013-2831

[2] Andersen SL, Olsen J, Wu CS, Laurberg P. Severity of birth defects after propylthiouracil exposure in early pregnancy. Thyroid. 2014;24(10):1533‐1540. 24963758 10.1089/thy.2014.0150 PMC4195247

[3] Nguyen CT, Sasso EB, Barton L, Mestman JH. Graves’ hyperthyroidism in pregnancy: a clinical review. Clin Diabetes Endocrinol. 2018;4(1):4. 29507751 10.1186/s40842-018-0054-7 PMC5831855

[4] Andersen SL, Olsen J, Laurberg P. Antithyroid drug side effects in the population and in pregnancy. J Clin Endocrinol Metab. 2016;101(4):1606‐1614. 26815881 10.1210/jc.2015-4274

[5] Maneros AG, Beck AE, Turner EH, et al Mutations in KCTD1 cause scalp-ear-nipple syndrome. Am J Hum Genet. 2013;92(4):621‐626. 23541344 10.1016/j.ajhg.2013.03.002 PMC3617379

[6] Van Dijke CP, Heydendael RJ, De Kleine MJ. Methimazole, carbimazole, and congenital skin defects. Ann Intern Med. 1987;106(1):60‐61. 3789581 10.7326/0003-4819-106-1-60

[7] Mandel SJ, Brent GA, Larsen PR. Review of antithyroid drug use during pregnancy and report of a case of aplasia cutis. Thyroid. 1994;4(1):129‐133. 7519913 10.1089/thy.1994.4.129

[8] Taylor PN, Vaidya B. Side effects of anti-thyroid drugs and their impact on the choice of treatment for thyrotoxicosis in pregnancy. Eur Thyroid J. 2012;1(3):176‐185. 24783017 10.1159/000342920 PMC3821480

[9] Bowman P, Vaidya B. Suspected spontaneous reports of birth defects in the UK associated with the use of carbimazole and propylthiouracil in pregnancy. J Thyroid Res. 2011;2:235130. 10.4061/2011/235130 PMC3172977 21922050

[10] Clementi M, Di Gianantonio E, Cassina M, et al Treatment of hyperthyroidism in pregnancy and birth defects. J Clin Endocrinol Metab. 2010;95(11):E337‐E341. 20668039 10.1210/jc.2010-0652

